# Mysida and Lophogastrida of Greece: a preliminary checklist

**DOI:** 10.3897/BDJ.4.e9288

**Published:** 2016-11-01

**Authors:** Panayota Koulouri, Vasilis Gerovasileiou, Nicolas Bailly

**Affiliations:** ‡ Institute of Marine Biology, Biotechnology and Aquaculture, Hellenic Centre for Marine Research, Heraklion, Greece

**Keywords:** Mysida, Lophogastrida, Greece, Aegean Sea, Sea of Crete, Ionian Sea, Eastern Mediterranean, checklist

## Abstract

**Background:**

The checklist of Mysida and Lophogastrida of Greece was created within the framework of the Greek Taxon Information System (GTIS), which is one of the applications of the LifeWatchGreece Research Infrastructure (ESFRI) resuming efforts to develop a complete checklist of species recorded and reported from Greek waters. The objectives of the present study were to update and cross-check taxonomically all records of Mysida and Lophogastrida species known to occur in Greek waters in order to search for inaccuracies and omissions.

**New information:**

The up-to-date checklist of Mysida and Lophogastrida of Greece comprises 49 species, classified to 25 genera.

## Introduction

The peracarid crustaceans Lophogastrida, Stygiomysida and Mysida were formerly grouped under the order "Mysidacea". Although these three monophyletic groups are now ranked as distinct orders of Peracarida (Meland and Willassen 2007, Meland et al. 2015), we have chosen to present mysids and lophogastrids under the same checklist, while no stygiomysids have been recorded so far from Greek waters. The most important contribution to the Greek records of the taxonomic group of "Mysidacea" has been made by Hatzakis (Hatzakis 1974, Hatzakis 1977, Hatzakis 1978, Hatzakis 1982) followed later on by Madurell and Cartes (2003) as well as Koulouri et al. (2013). "Mysidacea" constitute the dominant faunal component for the so-called hyperbenthos, which includes all swimming bottom-dependent organisms (mainly crustaceans) performing, with varying amplitude, intensity and regularity, seasonal or daily vertical migrations into the water column (Brunel et al. 1978). A large proportion of these animals are living within the benthic boundary layer (BBL) which is the portion of the sediment and the water column that is influenced by the sediment-water interface and characterized by strong gradients of flow as well as high concentrations of dissolved and particulate organic matter (Boudreau and Jørgensen 2001). Sampling these often highly mobile animals is not easy as they are caught occasionally by conventional benthic (e.g., grabs, corers) or pelagic (e.g. plankton nets) sampling gears (Eleftheriou 2013). Specially designed samplers, generally known as hyperbenthic or suprabenthic sledges, have been constructed, modified and used over the last 40 years for sampling these macrofaunal organisms, but there are still practical technical difficulties in the accessibility of this particular habitat (Mees and Jones 1997, Eleftheriou 2013, Koulouri et al. 2013). However, and when depth allows, these organisms can also be sampled by scuba diver operated "hunting" tools such as hand nets (e.g. Ledoyer 1968) or suction bottles (e.g. Chevaldonné et al. 2008).

A first attempt for creating a checklist of Mysida and Lophogastrida under the order of "Mysidacea" was carried out in the context of the “Greek Biodiversity Database” project (2005-2008), coordinated by the Department of Zoology, at the Aristotle University of Thessaloniki. The documented occurrence of these marine species in Greek waters was recorded in a database that was set up online in 2010. Although not completed, the database had not been updated since the closure of the project (2010). During the European project PESI, WoRMS/ERMS specially created the reference Koukouras (2010) for the list of marine species provided by the Greek Biodiversity Database. The aim of the present work was to present an updated checklist of Lophogastrida and Mysida of the Greek waters. For this purpose, older lists were updated and annotated according to the recent literature and current taxonomic status of the species.

## Materials and methods

The Checklist of Mysida and Lophogastrida of Greece (Suppl. material 1​) was created within the framework of the Greek Taxon Information System (GTIS), one of the applications of the LifeWatchGreece Research Infrastructure (ESFRI), aiming at developing a complete checklist of all species reported from Greek waters (Bailly et al. 2016, this special collection). The general principles used for elaborating this Preliminary Checklist are given in Bailly et al. (2016). The checklist of Mysida and Lophogastrida of Greece was constructed based on the species records extracted from the dataset of WoRMS/ERMS for marine species. Then, all recent publications were reviewed and the species reported to date have been added to the list. The classification followed in the present checklist is the one proposed by the World List of Lophogastrida, Stygiomysida and Mysida (Mees and Meland 2012).

## Checklists

### Checklist of Mysida and Lophogastrida known to occur in Greek waters

#### Lophogastrida

#### Eucopiidae

#### Eucopia unguiculata

(Willemoes-Suhm, 1875)

#### Lophogastridae

#### Lophogaster typicus

M. Sars, 1857

#### Mysida

#### Mysidae

#### Acanthomysis longicornis

(Milne Edwards, 1837)

#### Anchialina agilis

(G.O. Sars, 1877)

#### Anchialina oculata

Hoenigman, 1960

#### Boreomysis arctica

(Krøyer, 1861)

#### Boreomysis megalops

G.O. Sars, 1872

#### Calyptomma puritani

W. Tattersall, 1909

#### Dactylamblyops corberai

San Vicente & Cartes, 2011

#### Diamysis bacescui

Wittmann & Ariani, 1998

#### Diamysis cymodoceae

Wittmann & Ariani, 2012

#### Diamysis lagunaris

Ariani & Wittmann, 2000

#### Diamysis mesohalobia

Ariani & Wittmann, 2000

#### Erythrops elegans

(G.O. Sars, 1863)

#### Erythrops erythrophthalmus

(Goës, 1864)

#### Erythrops neapolitanus

Colosi, 1929

#### Euchaetomeropsis merolepis

(Illig, 1908)

#### Gastrosaccus mediterraneus

Bacescu, 1970

#### Gastrosaccus sanctus

(Van Beneden, 1861)

#### Haplostylus bacescui

Hatzakis, 1977

#### Haplostylus lobatus

(Nouvel, 1951)

#### Haplostylus normani

(G.O. Sars, 1877)

#### Harmelinella mariannae

Ledoyer, 1989

#### Hemimysis margalefi

Alcaraz, Riera & Gili, 1986

#### Heteromysis eideri

Bacescu, 1941

#### Leptomysis buergii

Bacescu, 1966

#### Leptomysis gracilis

(G.O. Sars, 1864)

#### Leptomysis lingvura

(G.O. Sars, 1866)

#### Leptomysis mediterranea

G.O. Sars, 1877

#### Leptomysis megalops

Zimmer, 1915

#### Leptomysis truncata

(Heller, 1863)

#### Limnomysis benedeni

Czerniavsky, 1882

#### Mesopodopsis aegyptia

Wittmann, 1992

#### Mesopodopsis slabberi

(Van Beneden, 1861)

#### Mysideis parva

Zimmer, 1915

#### Mysidopsis angusta

G.O. Sars, 1864

#### Mysidopsis gibbosa

G.O. Sars, 1864

#### Paraleptomysis apiops

(G.O. Sars, 1877)

#### Paramysis agigensis

Bacescu, 1940

#### Paramysis helleri

(G.O. Sars, 1877)

#### Paramysis kosswigi

Bacescu, 1948

#### Parerythrops paucispinosus

Nouvel & Lagardère, 1976

#### Pseudomma nanum

Holt & Tattersall, 1906

#### Siriella armata

(Milne Edwards, 1837)

#### Siriella clausii

G.O. Sars, 1877

#### Siriella gracilipes

Nouvel, 1942

#### Siriella jaltensis

Czerniavsky, 1868

#### Siriella norvegica

G.O. Sars, 1869

#### Siriella thompsonii

(H. Milne Edwards, 1837)

## Discussion

A total of 49 species, classified in 25 genera, makes up the updated checklist of Lophogastrida and Mysida of Greece. More specifically, the order of Lophogastrida comprises 2 species classified to 2 genera, while the order of Mysida comprises 47 species, classified to 23 genera. It is worth mentioning that *Dactylamblyops corberai* San Vicente & Cartes, 2011, a new mysid species was described from the deep Ionian Sea by San Vicente and Cartes (2011), revealing the limited sampling effort that has taken place for these particular taxa. It should also be noted that Chevaldonné et al. (2015) provided new molecular and distribution data on the poorly known cave mysid *Harmelinella mariannae* Ledoyer, 1989. A number of issues aroused during the thorough examination of the checklist of Mysida of Greece, specifically regarding the presence of the species *Diamysis bahirensis* (Sars G.O., 1877), *Haplostylus magnilobatus* (Bacescu & Schiecke, 1974) and *Paramysis festae* Colosi, 1921 in Greek waters. The material previously indicated as *D. bahirensis* by Hatzakis (1982) has been revised and assigned to *D. lagunaris* and *D. bacescui* (Wittmann and Ariani 1998, Wittmann and Ariani 2012). Therefore, further research is needed to verify the presence of this species in the Greek waters. The species *H. magnilobatus* has been included in the mysid species of the Aegean Sea according to Coll et al. (2010). However, the reference used for this record does not actually include this species in the Greek fauna (Hatzakis 1977). The species *P. festae* has been recorded in the Ionian coast of Sicily and in a brackish lagoon of Libya (Audzijonyte et al. 2008, Wittmann and Ariani 2011), but not yet in Greek waters. Moreover, further research is needed for the verification of the presence of the mysid species recorded by Koulouri et al. (2013) as Erythrops cf. peterdorhni Basescu & Schiecke, 1974 in the Greek waters. We should also note that when establishing the new rank *Siriella gracilipes* for part of what was called *S. jaltensis*, Wittmann and Wirtz (1998) reported the finding of *S. gracilipes* in the Aegean Sea. Finally, two provisional cryptic species of the complex taxon *Hemimysis margalefi* *sensu lato* were recorded from marine cave habitats from Corfu and Crete islands by using molecular techniques (Rastorgueff et al. 2014) and therefore, their taxonomic status needs to be further studied. As far as eastern Mediterranean is concerned, Mysida and Lophogastrida have received little attention mostly due to difficulties in sampling these particular taxa especially in oligotrophic shelf areas (Koulouri et al. 2013). However, it is expected that several species distributed in other Mediterranean regions will be recorded as elements of the Greek fauna in the future.

## Supplementary Material

Supplementary material 1 Checklist of Mysida and Lophogastrida (Arthropoda: Malacostraca) of Greece Data type: Taxonomic checklist
Brief description: Checklist of Mysida and Lophogastrida known to occur in Greek waters.
File: oo_97965.xls Panayota Koulouri, Vasilis Gerovasileiou, Nicolas Bailly

XML Treatment for Lophogastrida

XML Treatment for Eucopiidae

XML Treatment for Eucopia unguiculata

XML Treatment for Lophogastridae

XML Treatment for Lophogaster typicus

XML Treatment for Mysida

XML Treatment for Mysidae

XML Treatment for Acanthomysis longicornis

XML Treatment for Anchialina agilis

XML Treatment for Anchialina oculata

XML Treatment for Boreomysis arctica

XML Treatment for Boreomysis megalops

XML Treatment for Calyptomma puritani

XML Treatment for Dactylamblyops corberai

XML Treatment for Diamysis bacescui

XML Treatment for Diamysis cymodoceae

XML Treatment for Diamysis lagunaris

XML Treatment for Diamysis mesohalobia

XML Treatment for Erythrops elegans

XML Treatment for Erythrops erythrophthalmus

XML Treatment for Erythrops neapolitanus

XML Treatment for Euchaetomeropsis merolepis

XML Treatment for Gastrosaccus mediterraneus

XML Treatment for Gastrosaccus sanctus

XML Treatment for Haplostylus bacescui

XML Treatment for Haplostylus lobatus

XML Treatment for Haplostylus normani

XML Treatment for Harmelinella mariannae

XML Treatment for Hemimysis margalefi

XML Treatment for Heteromysis eideri

XML Treatment for Leptomysis buergii

XML Treatment for Leptomysis gracilis

XML Treatment for Leptomysis lingvura

XML Treatment for Leptomysis mediterranea

XML Treatment for Leptomysis megalops

XML Treatment for Leptomysis truncata

XML Treatment for Limnomysis benedeni

XML Treatment for Mesopodopsis aegyptia

XML Treatment for Mesopodopsis slabberi

XML Treatment for Mysideis parva

XML Treatment for Mysidopsis angusta

XML Treatment for Mysidopsis gibbosa

XML Treatment for Paraleptomysis apiops

XML Treatment for Paramysis agigensis

XML Treatment for Paramysis helleri

XML Treatment for Paramysis kosswigi

XML Treatment for Parerythrops paucispinosus

XML Treatment for Pseudomma nanum

XML Treatment for Siriella armata

XML Treatment for Siriella clausii

XML Treatment for Siriella gracilipes

XML Treatment for Siriella jaltensis

XML Treatment for Siriella norvegica

XML Treatment for Siriella thompsonii
